# Numerical Modeling and Computer Simulation of a Meander Line Antenna for Alzheimer’s Disease Treatment, a Feasibility Study

**DOI:** 10.4236/jbm.2023.112013

**Published:** 2023-02

**Authors:** Felipe P. Perez, Maryam Rahmani, Jorge Morisaki, Farhan Amran, Syazwani Bakri, Akmal Halim, Alston Dsouza, Nurafifi Mohd Yusuff, Amran Farhan, James Maulucci, Maher Rizkalla

**Affiliations:** 1 Department of Medicine, Division of General Internal Medicine and Geriatrics, Indiana University School of Medicine, Indianapolis, USA; 2 Department of Electrical and Computer Engineering, Indiana University-Purdue University, Indianapolis, USA; 3 Department of Bioengineering, University of Illinois at Chicago, Chicago, USA

**Keywords:** Alzheimer’s Disease, Meander Line Antenna, HFSS, EMF Linearity, SAR, Field Distribution

## Abstract

Alzheimer’s disease (AD) is a brain disorder that eventually causes memory loss and the ability to perform simple cognitive functions; research efforts within pharmaceuticals and other medical treatments have minimal impact on the disease. Our preliminary biological studies showed that Repeated Electromagnetic Field Stimulation (REFMS) applying an EM frequency of 64 MHz and a specific absorption rate (SAR) of 0.4 – 0.9 W/kg decrease the level of amyloid-*β* peptides (A*β*), which is the most likely etiology of AD. This study emphasizes uniform E/H field and SAR distribution with adequate penetration depth penetration through multiple human head layers driven with low input power for safety treatments. In this work, we performed numerical modeling and computer simulations of a portable Meander Line antenna (MLA) to achieve the required EMF parameters to treat AD. The MLA device features a low cost, small size, wide bandwidth, and the ability to integrate into a portable system. This study utilized a High-Frequency Simulation System (HFSS) in the design of the MLA with the desired characteristics suited for AD treatment in humans. The team designed a 24-turn antenna with a 60 cm length and 25 cm width and achieved the required resonant frequency of 64 MHz. Here we used two numerical human head phantoms to test the antenna, the MIDA and spherical head phantom with six and seven tissue layers, respectively. The antenna was fed from a 50-Watt input source to obtain the SAR of 0.6 W/kg requirement in the center of the simulated brain tissue layer. We found that the E/H field and SAR distribution produced was not homogeneous; there were areas of high SAR values close to the antenna transmitter, also areas of low SAR value far away from the antenna. This paper details the antenna parameters, the scattering parameters response, the efficiency response, and the E and H field distribution; we presented the computer simulation results and discussed future work for a practical model.

## 1. Introduction

Small size antennas have been the focus of cellular communication systems, bioinstrumentations, medical and IoT devices. This is due to high demand of the compact equipment. The small size antennas in medical applications are suited for use in and around human bodies. There are multiple antenna approaches [1] [2] [3] [4] for the E/H linearity within the human body, while lacks the portable size feature that enables integrating them around the human tissue sizes. The rapid development in devices that emit Radio Frequency Electromagnetic Fields (RF-EMF) has sparked a growing interest in their interaction with biological systems and their beneficial effects on human health. As a result, investigations are motivated by the potential for therapeutic applications and concern for any possible side effects of these EM energies. Some studies have indicated that a specific tuning of experimental and clinical Radio Frequency (RF) exposure may lead to their clinical application toward beneficial health outcomes.

**Figure 1.**
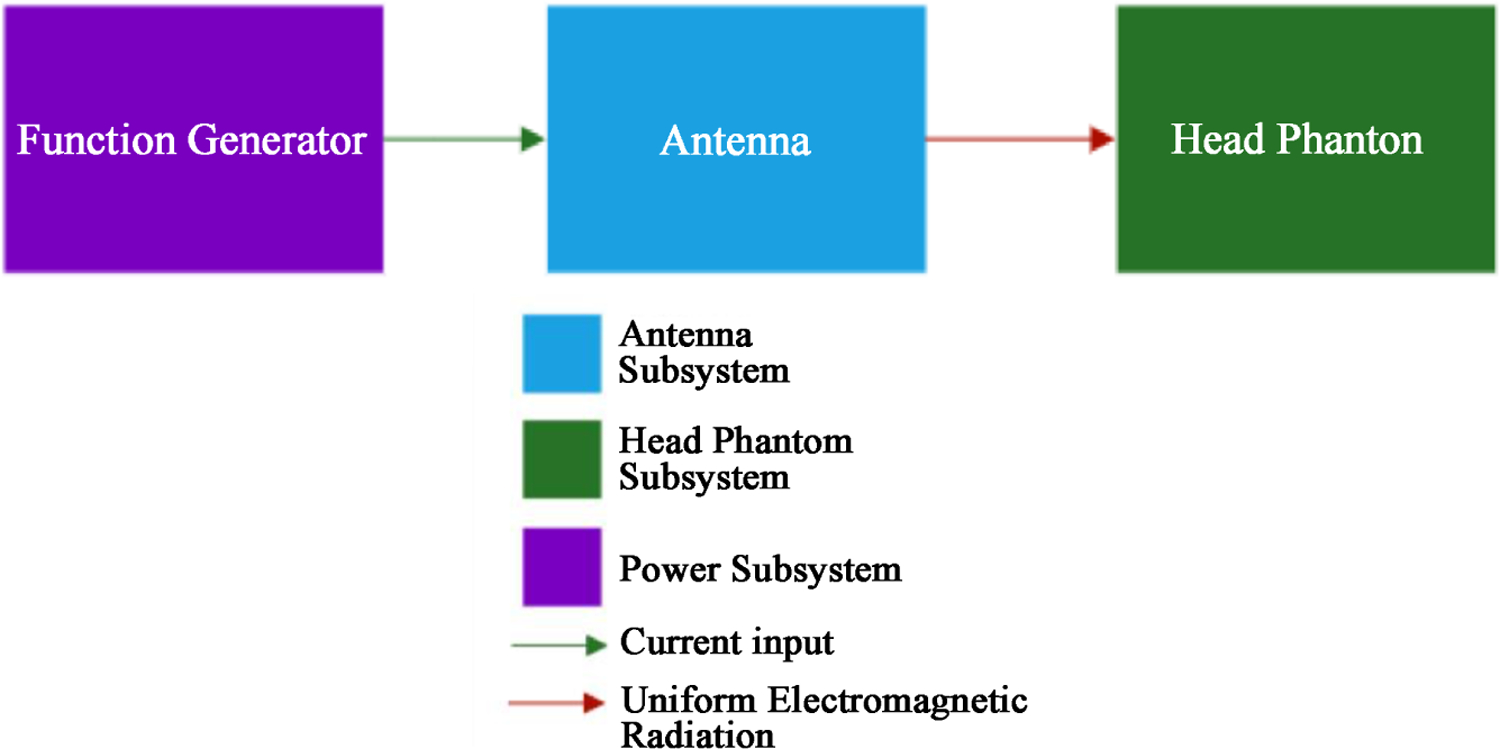
System block diagram.

Figure 1 shows the block diagram of the proposed system. The function generator will provide the EM power required to transmit the appropriate SAR in the brain layer. The antenna will radiate and focus the EM energy in the human head phantom direction. The antenna position will be on top of the head phantom. The head phantom will simulate a real human head with 5 tissue layers, EMF exposure will reach a SAR of 0.4 – 0.9 W/kg in the brain layer.

The application investigated here is the near zone field distribution for HF/VHF antenna since the human head is placed very close to the antenna surface. The study of near zone field has been investigated and measured for their linear distribution [5] [6]. The size of the antenna to perform at 64 MHZ (VHF) is important for wearable devices. The design has been implemented for 64 MHZ while the size of the antenna was beyond the wearing device limitation [7].

## 2. Antenna Design

**Figure 2.**
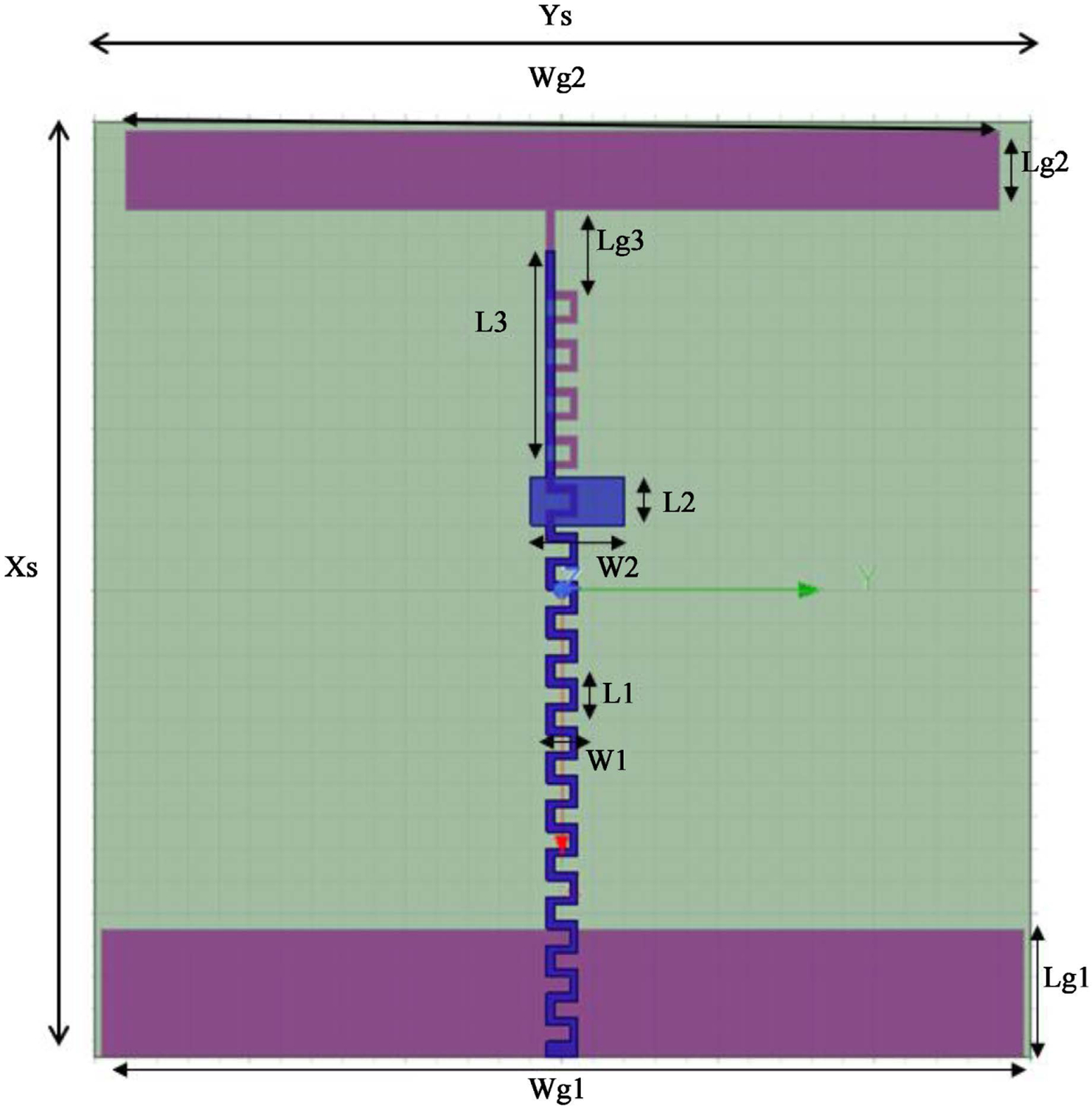
Geometry of the meander monopole line antenna.

We chose the Meander Line Antenna (MLA) for this design [8] [9], because the antenna size is suitable for a portable system and satisfies the AD treatment SAR requirements. Figure 2 shows the geometry, and Table 1 shows the detailed dimensions of the antenna.

Table 2 shows the dimensions of the various layers within the human head phantom and the dielectric properties of each layer. The parameters in Table 2 were incorporated into the HFSS simulation system for the antenna radiation at the given frequency. The material of the substrate is Rogers RO30130 which has a high dielectric value of 10.2 that allows the antenna to obtain a higher gain within the human head phantom direction. The number of meander turns also affects the radiation frequency. The number of turns for the meander line of ground is 27 turns and 21 turns for the top patch.

## 3. The Head Phantom Models

In this study, we performed two different simulation head phantom models, the Multimodal Imaging-Based Detailed Anatomical Model of the Human Head and Neck (MIDA), and the Spherical Model Head Phantom.

Both head phantoms were based on the parameters stated in Table 2.

## 4. Results and Discussion

### 4.1. Antenna and MIDA Head Phantom

**Figure 3.**
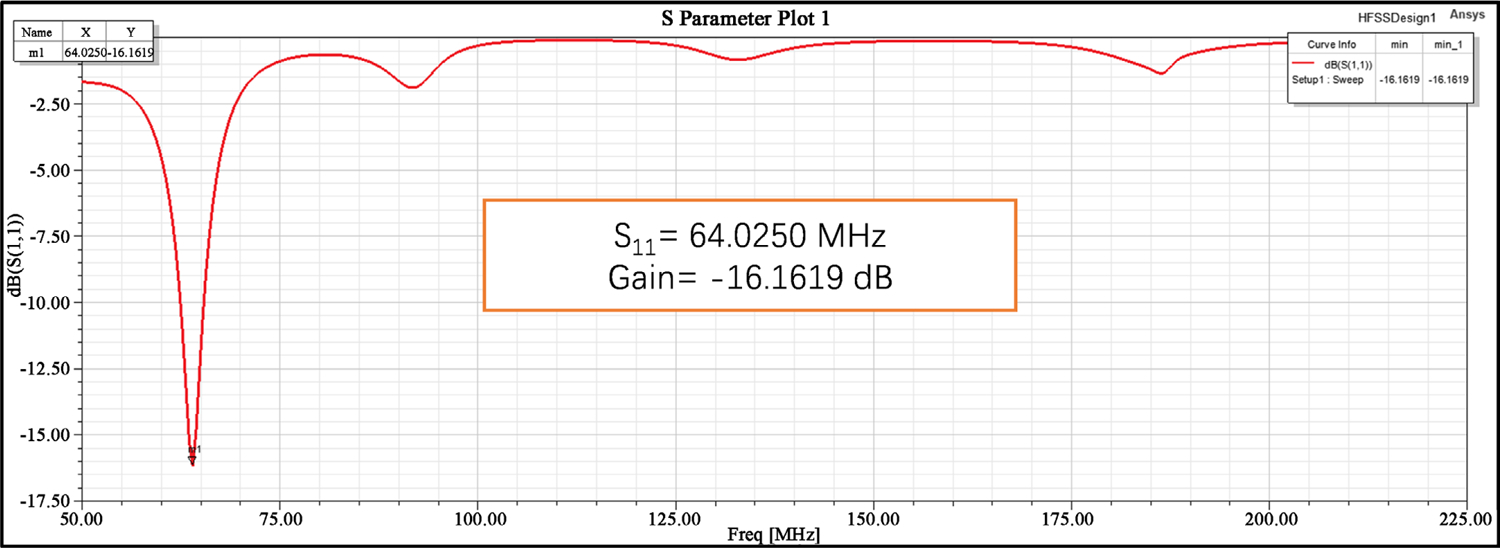
S_11_ Parameter VS gain graph of the antenna.

**Figure 4.**
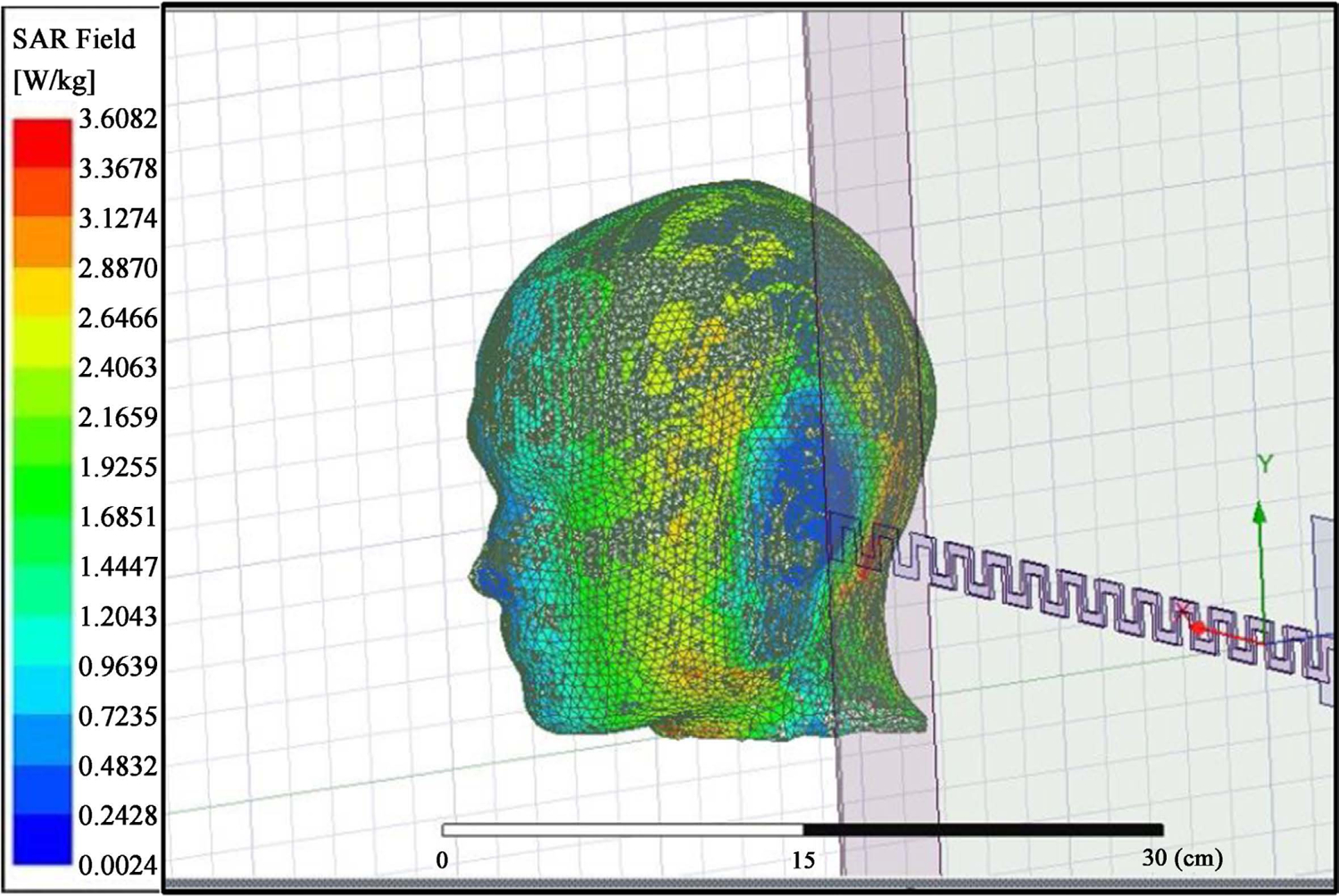
SAR result on the MIDA human head phantom.

Figure 3 shows the Scattering S_11_ parameters of the resonating frequency of 64 MHz, and Figure 4 shows the SAR distribution in the MIDA human head phantom. Table 3 shows the minimum and maximum SAR observed in the various layers before reaching the brain layer; the SAR in the brain layer in blue color was between 0.9 and 0.0024 W/kg. We observed that the nonlinearity was an issue with this antenna exposure on the human head phantom model.

Within the five different layers of surfaces simulated for the SAR values for the MIDA [10] head phantom, the skin appeared to have a very high average SAR value because it is the closest surface to the antenna radiation. We observed that the power was dissipated in the skin before the radiation reached the skull. The values of the SAR across the head phantom are relatively not linear. One possible cause is that the source of the EMF radiation is on top of the head and the SAR decreases with increasing distance from the antenna. The other cause is that the SAR is calculated based on the mass density of each layer and not on the whole head. This causes some errors in the SAR value and produces a non-homogeneous distribution of the SAR values in each layer. For example, the SAR values in the brain layer are between 0.0024 and 0.9639 W/kg, therefore showing areas of the brain with insufficient energy to produce biological effects. The inconsistency of the SAR values is also due to the complex structure of the MIDA head phantom with complex boundaries between layers.

### 4.2. Antenna and Spherical Model Head Phantom

**Figure 5.**
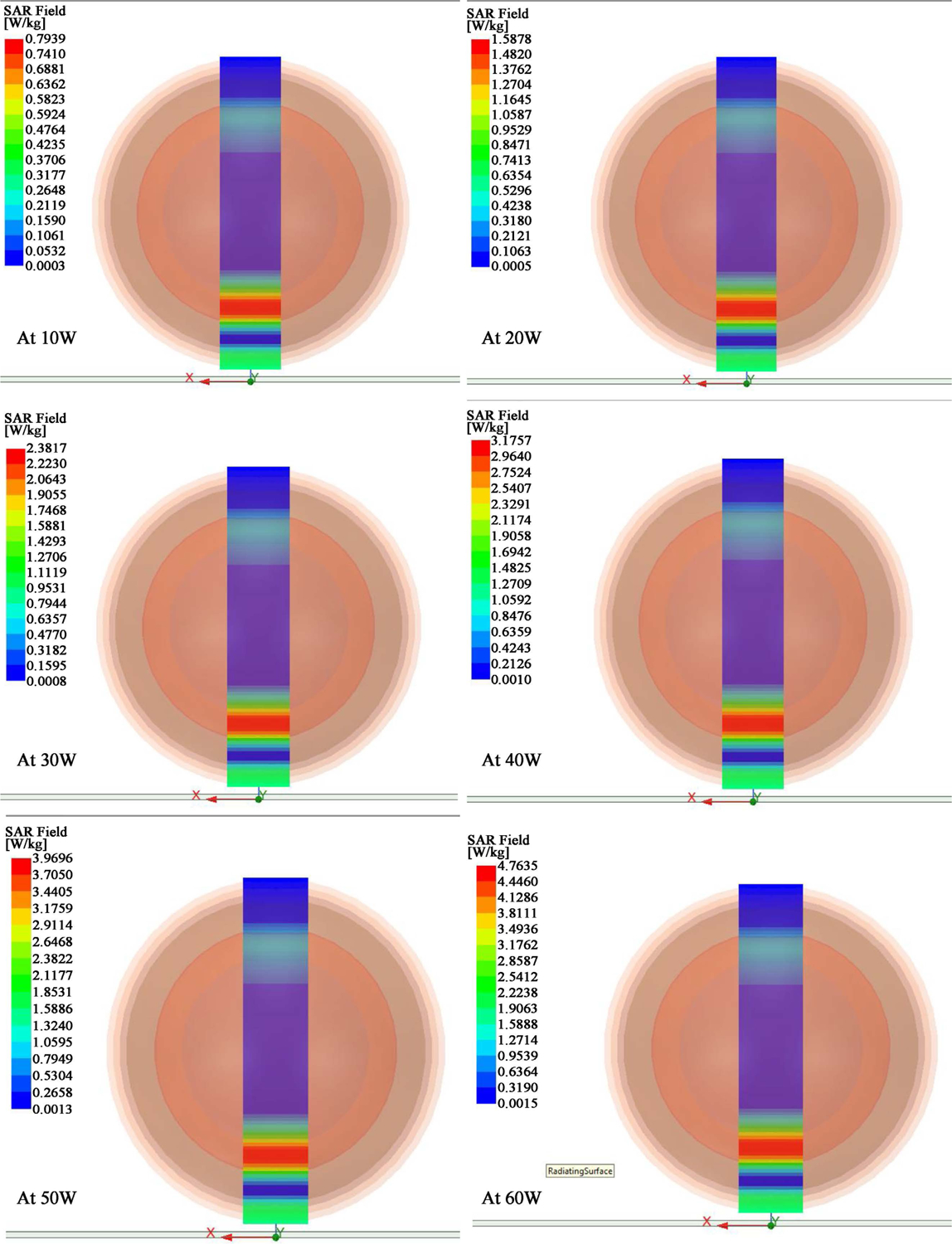
SAR field with various power applied. We determined that the 30 W, 40 W, 50 W and 60 W produced a SAR between 0.4 and 0.9 W/kg (see Table 4) in the center of the simulated brain layer (in purple color).

We utilized the same MLA antenna to perform EMF exposures to the spherical model head phantom with the S11 parameters. We tried several power levels until we reached the appropriate SAR of 0.4 – 0.9 W/kg. Figure 5 gives the SAR distribution for two different power values; 1 W and 50 W. Table 4 shows the SAR versus power generated and fed into the antenna. We reached a SAR of 0.6 W/kg at the brain tissue of the human head phantom using a power of 50 W.

The average SAR is higher in the layers near the antenna and decreases as the distance increases. These results were expected as the tissues absorbed the energy of the radiation emitted by the antenna. When the power of the antenna is manipulated, we can observe that the distribution pattern of the average SAR is kept constant on each layer of the tissue.

## 5. Conclusion & Future Work

In this work, we have demonstrated that the MLA antennas are suitable for portable devices and for producing a SAR of 0.4 – 0.9 W/kg in the simulated human brain within reasonable dimensions. The study was based on a near-field distribution. The HFSS simulation showed a scattering parameter S_11_ at 64 MHZ while keeping the antenna size within the portable range. The two head phantom models studied here show that the spherical model gives accurate data and better linearity; this is caused by the complex mathematical model of the MIDA phantom and the software capacity to handle it. Here we also determined that the SAR values decreased in tissues farthest from the antenna transmitter and produced a non-homogeneous distribution pattern which is not appropriate for future AD treatments unless we use two or more antennas to obtain a more homogeneous SAR distribution. A practical model is needed to confirm the HFSS data model, we will reserve the practical model for future consideration [11] [12].

## Figures and Tables

**Table 1.** Dimensions of the meander monopole line antenna.

**Xs**	58.0 cm	**Wg2**	56.0 cm	**L2**	3.0 cm
**Ys**	60.0 cm	**Lg2**	5.0 cm	**W2**	6.0 cm
**Wg1**	59.0 cm	**L1**	2.0 cm	**L3**	14.0 cm
**Lg1**	8.0 cm	**W1**	2.0 cm	**Lg3**	7.0 cm

**Table 2.** Properties of the Seven Different Layers of the Brain at a frequency of 64 MHz.

Layer	Radius (mm)	Permittivity (*e*_*r*_)	Permeability	Conductivity (S/m)	Mass Density (kg/m^3^)
Skin	51.0	32	1	0.1483	1109.0
Fat	49.0	9	1	0.0776	0911.0
Muscle	47.0	50	1	0.4466	1090.4
Skull	45.0	10	1	0.0179	1908.0
Dura	36.5	40	1	0.0600	1174.0
CSF	36.0	65	1	1.8790	1007.0
Brain	28.1	40	1	0.6165	1045.5

**Table 3.** Tabulated data of average SAR in the different layers of the MIDA human head phantom.

Layers	Max. Value of Average SAR (W/kg)	Min. Value of Average SAR (W/kg)
Skin	3.6082	0.0264
Skull	2.8870	0.0261
Dura	1.2043	0.0104
CSF	2.1669	0.0303
Brain	0.9639	0.0024

**Table 4.** Average SAR in the simulated brain layer. We increased the power to 50 W to reach a SAR of 0.63 – 0.95 W/kg in the simulated brain.

Power (W)	Average SAR (W/kg)	Median Value (W/kg)
1	0.0113 – 0.0170	0.01415
10	0.1061 – 0.1590	0.13255
20	0.2121 – 0.3180	0.26505
30	0.3182 – 0.4770	0.39760
40	0.4243 – 0.6359	0.53010
50	0.5304 – 0.7929	0.66165
60	0.6364 – 0.9539	0.79515
70	0.7425 – 1.1129	0.9277
80	0.8486 – 1.2719	1.06025
90	0.9437 – 1.4309	1.1873
100	1.0607 – 1.5898	1.32525
